# Gut Microbiota: Therapeutic Targets of Ginseng Against Multiple Disorders and Ginsenoside Transformation

**DOI:** 10.3389/fcimb.2022.853981

**Published:** 2022-04-25

**Authors:** Zhaoqiang Chen, Zepeng Zhang, Jiaqi Liu, Hongyu Qi, Jing Li, Jinjin Chen, Qingxia Huang, Qing Liu, Jia Mi, Xiangyan Li

**Affiliations:** ^1^ Jilin Ginseng Academy, Key Laboratory of Active Substances and Biological Mechanisms of Ginseng Efficacy, Ministry of Education, Jilin Provincial Key Laboratory of Bio-Macromolecules of Chinese Medicine, Changchun University of Chinese Medicine, Changchun, China; ^2^ Research Center of Traditional Chinese Medicine, The First Affiliated Hospital of Changchun University of Chinese Medicine, Changchun, China; ^3^ College of Acupuncture and Tuina, Changchun University of Chinese Medicine, Changchun, China; ^4^ Department of Endocrinology, The First Affiliated Hospital of Changchun University of Chinese Medicine, Changchun, China

**Keywords:** ginseng (*Panax ginseng C.A. Meyer*), gut microbiota, multiple disorders, gisenoside transformation, ginsenoside

## Abstract

Panax ginseng, as the king of Chinese herb, has significant therapeutic effects on obesity, type 2 diabetes mellitus, fatty liver disease, colitis, diarrhea, and many other diseases. This review systematically summarized recent findings, which show that ginseng plays its role by regulating gut microbiota diversity, and gut microbiota could also regulate the transformation of ginsenosides. We conclude the characteristics of ginseng in regulating gut microbiota, as the potential targets to prevent and treat metabolic diseases, colitis, neurological diseases, cancer, and other diseases. Ginseng treatment can increase some probiotics such as *Bifidobacterium*, *Bacteroides*, *Verrucomicrobia*, *Akkermansia*, and reduce pathogenic bacteria such as *Deferribacters*, *Lactobacillus*, *Helicobacter* against various diseases. Meanwhile, *Bacteroides*, *Eubacterium*, and *Bifidobacterium* were found to be the key bacteria for ginsenoside transformation *in vivo*. Overall, ginseng can regulate gut microbiome diversity, further affect the synthesis of secondary metabolites, as well as promote the transformation of ginsenosides for improving the absorptivity of ginsenosides. This review can provide better insight into the interaction of ginseng with gut microbiota in multiple disorders and ginsenoside transformation.

## Introduction

*Panax ginseng C. A. Meyer* (ginseng), called the king of herbs, has become one of the most popular Chinese herbal medicines in the world. It is widely used to treat various diseases (Ratan et al., 2021) and regulate human health (Fan et al., 2020). Moreover, ginseng also has high production value. According to statistics in 2016, China’s Ginseng output reached 28,900 tons, creating an output value of $7.5 billion for the ginseng industry in Jilin Province, ranking the first position (Li et al., 2019). In recent years, a variety of active ingredients have been isolated and identified from ginseng, mainly ginsenoside, polysaccharide, volatile oil, amino acid, polyacetylene, alkaloid, as well as a small amount of salicylic acid amine, organic acid, non-saponin water-soluble glucoside, maltol and its derivative glucoside (Liu et al., 2020). Polysaccharides, as one of the main components of ginseng, have the functions of immunomodulation, anti-tumor and anti-diabetes (Zhao et al., 2019). Amino acids in ginseng can enhance immunity, promote cell growth, proliferation and angiogenesis (Sah et al., 2021). Polyacetylene can significantly inhibit the proliferation of cancer cells and lipid peroxidation, improve memory (Al-Hazmi et al., 2015) and anti-inflammation (Jin et al., 2021). Alkaloids have the functions of radiation protection, anti-diabetic (Chen et al., 2019) and anti-tumor (Ajebli et al., 2021; Xu et al., 2021). The volatile oil in ginseng has the effects of antioxidation and liver protection (Bak et al., 2012). Ginsenoside is the most extensively studied active ingredient at this stage and has been considered as the main components of pharmacological action (Guo et al., 2021), such as anti-tumor (Kim et al., 2016), anti-inflammatory (Han and Kim, 2020), antioxidant, inhibition of apoptosis (Zheng et al., 2018).There are other trace components in ginseng, but they are not the main active components.

At present, it has been found that ginseng contains a variety of non-widespread initial ginsenosides, however, it does not have good biological activity, but after the processing and transformation of gut microbiota, it is found that there are 289 kinds of ginsenosides (including free ginsenoside) and their biological activities were significantly improved (Li et al., 2022). Although ginseng has many biological effects, the mechanisms of the pharmacological effect and its metabolic process *in vivo* have not been fully explained. In recent years, multiple studies have shown that the effect of ginseng is closely related to the role of gut microbiota (Pan et al., 2019; Santangelo et al., 2019), and gut microbiota is also the main tool for ginsenoside transformation (Kim, 2018).

Human microbial community is a complex ecosystem, in which the microbial community in the intestine has the largest scale and the most species (Tan et al., 2020). There are as many as 100 trillion microorganisms in human intestine, which are composed of at least 1,500 genera and 50 different phyla (Robles-Alonso and Guarner, 2013). In the long process of evolution of gut microbiota, through individual adaptation and natural selection, the microbiota of different species, microbiota and host, microbiota and host environment are always in a state of dynamic balance, forming an interdependent system. More and more evidences show that gut microbiota plays a key role not only in the metabolism of nutrients and drugs and the absorption of fat in the diet, but also in the regulation of immunity, physiology, metabolism, and health maintenance (Xi et al., 2021; Zhang et al., 2021). Besides, intestinal microorganisms could affect multiple tissues and organs, such as intestinal cells, liver, adipose tissue, brain, and muscle. These extensive studies about intestinal microbiota have made great progress in both humans and animals. As an example, specific changes in microbiota composition are associated with various diseases (Cani et al., 2021). For example, the ratio of *Firmicutes* to *Bacteroidetes* is increased in obese patients, while it is decreased in enteritis patients (Stojanov et al., 2020). Meanwhile, many treatments have targeted gut microbiota, which have found that probiotics are a potential treatment option for different diseases, such as Alzheimer’s disease (Ji and Shen, 2021). Therefore, gut microbiota has been considered as potential targets for the prevention and treatment of various diseases.

Currently, there are a few reviews for summarizing the regulation of ginseng on gut microbiota in a series of diseases and the biotransformation of ginsenosides by gut microbiota. In our review, we searched and summarized the recent publications that ginseng could treat various diseases through the regulation of gut microbiota, including obesity, type 2 diabetes mellitus (T2DM), liver diseases, diarrhea, colitis, and others. Moreover, we summarized the gut microbiota affected by ginseng in the treatment process, and the transformation process of ginsenosides under the action of gut microbiota. By the relevant reports on ginseng and gut microbiota, it reveals new potential targets, intestinal microbiota for ginseng in the prevention and treatment of a variety of diseases and provides new insights into the future research direction, intestinal microorganisms as potential targets of ginseng.

## Ginseng Treats Various Diseases by Regulating Gut Microbiota

### Obesity

Obesity is a global health problem (Safaei et al., 2021). Many chronic diseases associated with obesity, such as diabetes, cardiovascular disease and non-alcoholic fatty liver disease which are the main causes of human death (Hu et al., 2017). At present, the studies have found that gut microbiota is a necessary condition for the progress of obesity. Transplanting the microbiota of obese mice into healthy mice can induce obesity in healthy mice. At the same time, the activation and accumulation of fat by gut microbiota have been gradually revealed (Crovesy et al., 2020; Liu et al., 2021). Ginseng extract could also induce *Enterococcus faecalis* to produce unsaturated long-chain fatty acids and myristic acid has good effect on activating brown fat, promoting the production of beige fat, and reducing the body weight of obese mice (Quan et al., 2020). *Firmicutes* can promote energy absorption and fat accumulation (Orbe-Orihuela et al., 2018), and *Bacteroides* can regulate bile acid metabolism, reduce the level of inflammation, and inhibit fat accumulation in obese hosts. The ratio of *Firmicutes* to *Bacteroidetes* also plays an important role in the process of obesity (Gibiino et al., 2018). At present, ginseng extract, red ginseng extract, and the combination of ginsenoside Rb1 with salvianolic acid B have been proved to reduce weight and lipid by decreasing the ratio of *Firmicutes* to *Bacteroidetes* (Zhou et al., 2020; Bai et al., 2021; Lee et al., 2021). Meanwhile, ginseng treatment for 28 days can also up-regulate *Bacteroides, Parabacteroides*, and *Lactobacillus* probiotics to reduce the abundance of bacteria that can induce obesity, such as *Firmicutes*, *Deferribactes, Helicobacter* in obese hosts. Monascus fermented ginseng can reduce the ratio of *Firmicutes* to *Bacteroides* in the intestinal tract of HFD rats, further to decrease the relative levels of sterol regulatory element binding protein-2 (SREBP-2) and hydroxymethylglutaryl-CoA (HMG-CoA) reductase, and increase the expression of CYP7A1 to improve lipid level of total cholesterol (TC) in blood and liver against metabolic disorder (Zhao et al., 2021). The microbiota structure in obese hosts might be the pathogenic factor, but ginseng can help the body return to the normal metabolic level and lose weight by improving this unbalanced microbiota structure. In obesity animal models, *Firmicutes, Bacteroides, Lactobacillus*, and *Helicobacter* may be potential targets for ginseng in the treatment of obesity (**Table 1**). Meanwhile, relative studies have found ginseng could reduce the body weight, blood lipid, blood glucose, and inflammatory level, as well as can significantly improve the structure of gut microbiota in obese patients (**Table 2**) (Song et al., 2014; Seong et al., 2021).

**Table 1:** Summary of anti-obesity activities of ginseng by regulating gut microbiota and related pathways.

Conditions	Compounds/Extracts	Dose/Period	Models	Gut microbiota		Mechanisms	Refs.
				Up	Down		
Obesity	Ginseng extract	10 mg/kg; 56 days	db/db mice	Efa		Increases myristoleic acid, brown adipose tissue activation and beige fat formation to reduce adiposity.	(Quan et al., 2020)
Obesity	Water extract of red and white ginseng	5.5 ml/kg; 70 days	HFD-induced BALB/c mice	Lac, Bac, Para	Fir	Increases UCP1 and LCFAs levels to decrease body weight, LPS, IFN-γ, IL-1β, IL-6, IL-10.	(Zhou et al., 2020)
Obesity	KRG extract	235 mg/kg; 28 days	HFD-induced C57BL/6 mice	Ver, Akk, Par, Muc	Pro, Def, Lac, Hel, Bar, All, Osc	Up-regulates insulin and leptin levels to down-regulate body weight, fat, GLU, Insulin resistance, GOT, GPT.	(Lee et al., 2021)
Obesity	Rb1 and salvianolic acid B	Rb1 20 mg/kg + salvianolic acid B 100 mg/kg; 5 days	HFD-induced C57BL/6 mice	Fir, Bac, Cor, Adl	Hel, Muc, Def, Dor	Declines the serum GLU, TC, TG, LDL-C, FFA and reduces the BW.	(Bai et al., 2021)
Obesity	Monascus ruber fermented ginseng	200-400 mg/kg/d 28 days	HFD-induced SD rats	Bac, Pre	Fir, Mur	Decreases the relative expression levels of SREBP-2 and HMG-CoA reductase, TC contents in blood and liver, increases the expression level of CYP7A1 to improve lipid levels in blood and lipid metabolism disorders.	(Zhao et al., 2021)
T2DM	Ginsenoside T19	10-60 mg/kg; 42 days	HFD+STZ-induced C57BL/6 mice	Pro, Bac	Fir, Cop, Str, Rum, Ana, Ros, Aci	Decreases the levels of GLU, OGTT, ITT, TC, TG and LDL by increasing the expressions of GLUT4, PI3K, AKT, GSK3β and AMPK	(Xu et al., 2020)
T2DM	Ginsenoside Rg5	1.0 mg/mL; 28 days	db/db mice	Bac, Lac, All, Bar, Cop, Par	Fir, Clo, Hel, Fla, Pse, Dor, Ace, Bil, Ros, Sci	Inhibits the NF-κB pathway to decrease IL-6, IL-1β levels of serum, decreases the expression of TLR4 and increases the expression of Occludin, ZO-1, IκB-α. to decrease liver index, Glu.	(Wei et al., 2020)
T2DM	Red ginseng, Aronia, shiitake mushroom, nattokinase	0.5-1 g/kg; 84 days	HFD-induced SD rats	Bac, Des	Fir, Clo, Ery	Decreases GLU and insulin resistance by inhibiting islet B cell apoptosis and increasing bone mineral density.	(Yang et al., 2018)
T2DM	BuZangTongLuo decoction	5 g/kg/day; 21 days	HFD-induced C57BL/6J mice	Bac, Pro, Ver, Bif, Akk	Fir, Bla, Wei, Esc, Shi, Kur	Reduces the Water and food intake to control GLU.	(Zheng et al., 2020)
T2DM	Ginseng polysaccharides + Rb	Polysaccharides 0.2-1g/kg Rb 160 mg/kg; 30 days	HFD-induced Wister rats	Fir	Bac	Increases β-glucosidase activity to lower blood sugar levels.	(Li et al., 2018)

Bif, Bifidobacterium; Fae, Faecalibacterium; Bla, Blautia; Efa, Efaecalis; Lac, Lactobacillus; Fir, Firmicutes; Bac, Bacteroidetes; Para, Parabacteroides; Ver, Verrucomicrobia; Akk, Akkermansia; Muc, Mucispirillum; Pro, Proteobacteria; Def, Deferribacteres; Hel, Helicobacter; Bar, Barnesiella; All, Allistipes; Osc, Oscillibacter; Cor, Coriobacteriaceae; Adl, Adlercreutzia; Dor, Dor; Cop, Coprobacter; Par, Parasutterella; Clo, Clostridium; Fla, Flavonifractor; Pse, Pseudoflavonifractor; Ace, Acetatifactor; Bil, Bilophila; Ros, Roseburia; Sci, Scillibacter; Str, Streptococcus; Rum, Ruminococcus; Ana, Anaerotruncus; Ros, Roseburia; Aci, Acidobacteria; Des, Desulfovibrio; Ery, Erysipelothrix; Ver, Verrucomicrobia; Bla, Blautia; Wei, Weissella; Esc, Escherichia; Shi, Shigella; Kur, Kurthia; STZ, streptozocin.

**Table 2:** Summary of ginseng regulating gut microbiota in human models.

Conditions	Compounds/Extracts	Dose/Period	Models	Gut microbiota		Mechanisms	Refs.
				Up	Down		
Obesity	Ginseng extract	4g; 56 days	Obesity middle-aged Korean women	Bif, Fae, Bla		Reduces the BW, BMI and GLU, TC, TG of the serum.	(Song et al., 2014)
Metabolic syndrome	Korean red ginseng powder	6000 mg/day; 56 days	patients with metabolic syndrome	Bac	Fir, Pro	Improves metabolic syndrome by reducing systolic blood pressure, serum lipid metabolism markers TC and LDL levels and insulin resistance.	(Seong et al., 2021)
NAFLD	KRG powder capsules (including Rg1, Rb1, Rg3)	4.5 mg/g; 2 g/day; 28 days	Patients with nonalcoholic statohepatitis	Bif, Esc, Eub, Erys, Kle	Fir, Fae, Par, Meg, Dia	Reduces AST, ALT, TC, TG, γ-GT levels in the liver.	(Hong et al., 2021)

### Type 2 Diabetes Mellitus (T2DM)

T2DM is a complex disease, which is characterized by hyperglycemia and its complications mainly caused by insulin resistance (Del Prato and Chilton, 2018; Himanshu et al., 2020). In recent years, it has been found that the disorder of gut microbiota is an important factor in the occurrence and development of T2DM (Canfora et al., 2019). For example, the ratio of *Firmicutes* and *Bacteroidetes* is generally relatively high in the T2DM hosts (Perry et al., 2016). Current results have confirmed that ginseng plays a role in improving blood glucose level and insulin sensitivity in patients with T2DM (Gui et al., 2016). It proved that the treatment with ginsenoside T19 in C57BL/6 mice model induced by high-fat diet combined with streptozotocin could exert therapeutic effect for treating T2DM by reducing the relative abundance of pathogenic bacteria such as *Coprobacillus* and decreasing the ratio of *Firmicutes* and *Bacteroidetes*, as well as enhancing the relative abundance of probiotics such as *Bacteroidetes* (Xu et al., 2020). Ginsenoside Rg5 can also reverse the disordered gut microbiota, alleviate metabolic endotoxemia, repair intestinal barrier, inhibit nuclear factor kappaB (NF-κB) and other related inflammatory pathways to improve T2DM of Db/Db mice (Wei et al., 2020). Besides these ginsenosides, the combination of red ginseng, Aronia, Shiitake mushroom, and Nattokinase and Buzhong Tongluo decoction including *Astragalus membranaceus*, *Dioscorea hemsleyi*, *Salvia miltiorrhiza*, *Scrophularia ningpoensis*, *Ophiopogon japonicus*, *Panax ginseng*, *Fritillariae cirrhosae*, and *Whitmania pigra Whitman* have been proved to be able to up-regulate *Bacteroides, Lactobacillus, Akkermansia* probiotics, reduce the abundance of bacteria causing hyperglycemia, such as *Helicobacter*, *Flavonifractor*, and reduce the ratio of *Firmicutes* and *Bacteroidetes*, thus play their hypoglycemic effects (Yang et al., 2018; Zheng et al., 2020). In another disorder, metabolic syndrome, the powder from Korean red ginseng (KRG) has a good therapeutic effect, and its mechanism might be related to the improvement of the disorder of lipid metabolism (the reductions of TC and low-density lipoprotein, LDL) by increasing the relative abundance of *Bacteroides* and reducing the relative abundance of *Firmicutes* and *Proteobacteria* (Seong et al., 2021). Most studies show that ginseng can correct the abnormal metabolic level in the body by reversing the ratio of *Firmicutes* and *Bacteroidetes*. However, in the experiment of T2DM rat model treated with ginseng polysaccharide combined with Rb1, the ratio of *Firmicutes* and *Bacteroidetes* were increased after the treatment, which finally reduced the blood glucose level and increased β-glucosidase activity (Li et al., 2018). As same as most medical treatment, the ratio of *Firmicutes* and *Bacteroidetes* could be decreased, verse originally elevated in the pathological model. This change of gut microbiota could reduce the body’s energy intake and improve the level of glucose and lipid metabolism. However, this ratio was increased after treatment with polysaccharide and Rb1 in the above research. The original author did not make a targeted explanation. We speculate that it may be related to the state of the model, but the specific reason remains to be further revealed.

Therefore, the disordered gut microbiota is the inducer of T2DM, but ginseng can reverse the structures of disordered gut microbiota to restore the glucose metabolism and improve the disorder of glucose and lipid metabolism, ultimately inhibit the development of T2DM (**Table 1**).

### Fatty Liver Diseases

Non-alcoholic fatty liver disease (NAFLD), a gradually serious health problem worldwide, is a chronic and multi-factorial cause of liver disease (Younossi et al., 2016), which is often observed in patients with obesity, dyslipidemia and diabetes, and manifesting as hepatic steatosis (Polyzos et al., 2019). Pathogenic causes of fatty liver diseases include insulin resistance, hepatic lipid metabolism disorder, inflammation, genetics, and other factors (Friedman et al., 2018). In addition, the studies have found that the gut microbiota is able to influence hepatic lipid metabolism as well as the balance of pro- inflammatory and anti-inflammatory factors in the liver. Therefore, the gut microbiota has been a potential target for the prevention and treatment of NAFLD (Kolodziejczyk et al., 2019). Ginseng is widely used in metabolic diseases and also has a significant effect on the improvement of NAFLD (Roh et al., 2020; Yoon et al., 2021; Gu et al., 2021). Another study of ginseng extract treatment for fatty liver mice induced by high-fat diet demonstrated that ginseng could reverse the structure of disturbed gut microbiota to inhibit the levels of sterol regulatory element binding protein-1c (SREBP-1c), fatty acid synthetase (FAS), and acetyl-coenzyme a carboxylase 1 (ACC-1), and increase carnitine palmityl transferase 1A (CPT-1a) expression to alleviate hepatic lipid accumulation and suppress NF-κB/I-kappa-B (IκB) inflammasome pathway (Liang et al., 2021).

Alcoholic fatty liver disease is also a highly harmful chronic liver disease. And its occurrence is confirmed to be associated with the gut microbiota (Liu et al., 2018). The researches have proved that fermented ginseng can up-regulate probiotics such as *Bifidobacterium, Lactobacillus, Akkermansia* and down-regulate pathogenic bacteria such as *Peptostreptococcaceae, Colibacillus* that lead to metabolic disorders, which can alleviate the level of inflammation and improve the situation of alcoholic liver injury (Fan et al., 2019). The further deterioration of non-alcoholic fatty liver disease and alcoholic fatty liver disease could lead to hepatitis and even liver cancer (Ocker, 2020). In a dimethylnitrosamine induced-cancer mice, ginsenoside Rg3 combined with Fe@Fe_3_O_4_ nanoparticles can reduce the number of cancerous cells and prolong the survival time of mice with liver cancer by elevating the ratio of *Firmicutes* and *Bacteroidetes* or reversing the pathogenic gut microbiota structure (Ren et al., 2020). In summary, ginseng can treat the animal liver diseases models by reversing the imbalanced gut microbiota, improving liver metabolism, and restoring host homeostasis (**Table 3**). Moreover, in NAFLD patients, KRG could decrease the ratio of *Firmicutes* and *Bacteroidetes* to improve liver lipid metabolism, decreass serum TC and total triglyceride (TG) (**Table 2**) (Hong et al., 2021).

**Table 3:** Summary of anti-liver diseases activities of ginseng by regulating gut microbiota and related pathways.

Conditions	Compounds/Extracts	Dose/Period	Models	Gut microbiota		Mechanisms	Refs.
				Up	Down		
NAFLD	Ginseng extract	200 mg/kg	HFD-induced C57BL/6J male mice	Bac, Eps, Ver	Fir, Pro, Act	Inhibits the NF-κB/Iκβ pathway, and increases the expression of ZO-1, Occludin, CPT-1a to decrease the level of TC, TG, AST, ALT, LDL-C, TNF-α, IL-1β, IL-6, FAS, ACC-1 in serum and liver.	(Liang et al., 2021)
Alcoholic liver injury	Fermented ginseng	390 mg/kg/day; 56 days	Alcohol feeding- C57BL/6N	Bif, Lac, Akk, Rum, Eub, Bil, Deh, Sut, All, Osc, Dor	Pep, Col, Ent, Par	Reduces the TNF-α, IL-6, LPS, ALT, AST levels of serum to decrease the liver index.	(Fan et al., 2019)
Liver cancer	conjugate Fe@Fe _3_ O _4_ nanoparticles with ginsenoside Rg3 (NpRg3)	70 mg/kg; 490 days	Dimethylnitrosamine-induced C57BL/6 mice	Bac, Ver, Rum, Akk, Bar	Fir, Lach, Rik	Increases the lifetime and alleviate the pathological state of liver by inhibiting the proliferation of tumor cells.	(Ren et al., 2020)

Eub, Eubacterium; Erys, Erysipelotrichaceae; Kle, Klebsiella; Meg, Megamonas; Dia, Dialister; Eps, Epsilonbacteraeota; Act, Actinobacteria; Deh, Dehalobacterium; Sut, Suterella; All, Allobaculum; Osci, Oscillospira; Pep, Peptostreptococcaceae; Col, Colibacillus; Ent, Enterococcus; Lach, Lachnospiraceae; Rik, Rikenella.

### Diarrhea

Diarrhea is one of the leading diseases with the highest morbidity and mortality worldwide which is characterized by acute and infectious (Shankar and Rosenbaum, 2020). Prolonged diarrhea can lead to the consequences such as hypovolemia, electrolyte imbalance, malnutrition, and skin damage (Reintam Blaser et al., 2015). There are many causes of diarrhea, and commonly there are the disorders of bile acid metabolism (Hughes et al., 2021), viruses (Becker-Dreps et al., 2020), and *Escherichia coli* infection (Ahmed et al., 2013). Furthermore, recent studies have found that gut microbiota is an important pathogenic factor leading to diarrhea (Fan and Pedersen, 2021). While the occurrence of diarrhea could be prevented and improved by restoring the structures of disorganized gut microbiota (Gallo et al., 2016). Ginseng can alleviate diarrheal symptoms by modulating the gut microbiota structure. In the Kunming mice induced by 5-fluorouracil, a combination of total ginsenoside and volatiles of *Atractylodes Macrocephala* can decrease the abundances of *Bacteroides*, *Proteobacteria* and other bacteria and increase the abundances of *Firmicutes* and *Lactobacillus* to restore gut microbiota homeostasis for the improvement of diarrheal situation (Wang et al., 2019a). Moreover, the combination of ginseng polysaccharides and volatilized oil from *Atractylodes Macrocephala* could reduce the diarrhea index and ameliorate colonic lesions by restoring the structures of gut microbiota in the disease state (Wang et al., 2019b). In addition, fermented ginseng and ginseng polysaccharides have also been proved to have therapeutic effects on diarrhea by up-regulating *Firmicutes*, *Lactobacillus*, the ratio of *Firmicutes* and *Bacteroidetes*, and down-regulating *Proteobacteria*, and *Bacteroidetes* (Qi et al., 2019; Qu et al., 2021). Taken together, the active components from ginseng can restore intestinal homeostatic balance and improve water and salt metabolism to alleviate diarrhea (**Table 4**).

**Table 4:** Summary of anti-colitis and anti-diarrhea activities of ginseng by regulating gut microbiota and related pathways.

Conditions	Compounds/Extracts	Dose/Period	Models	Gut microbiota		Mechanisms	Refs.
				Up	Down		
Diarrhea	Atractylodes macrocephala volatile oil and total ginsenosides	Ginseng and Atractylodes 1:1 40 mg/kg/d; 42days	5-fluorouracil induced Kunming mice	Fir, Lac	Bac, Pro, Rum, Ana, Des	Reduces diarrhea index, thymus, and spleen index, improves the pathological changes of colon, increases body weight by reducing the content of TNF-α, IFN-γ, IL-6, IL-1β, IL-17 in serum	(Wang et al., 2019a)
Diarrhea	Shenzhu capsule (Contains ginseng)	Saponins and polysaccharides 126.7 mg/kg/day, 176.3 mg/kg/day, Atractylodes macrocephala volatile oil and polysaccharides 35.3μL/kg/day, 192.3mg/kg/day; 64days	Chemotherapy-induced Kunming mice	Fir, Lac, Clo, Des, Allo	Bac, Pro, Prevo	Improves the body weight by decreasing diarrhea rate, thymus spleen index and colonic pathological changes	(Wang et al., 2019b)
Diarrhea	Fermented ginseng	0.125-2 g/kg/day; 5days	SD rats	Lac, Bif, Ent, Bac		Inhibits the NF-κB pathway to reduce inflammation and inhibits the expression of. TLR4, increase weight by lowering the liver index	(Qu et al., 2021)
Diarrhea	Ginseng polysaccharide	100 mg/kg; 7days	Lincomycin hydrochloride-induced Balb/c mice	Fir, Act, Lac, Str	Pro, Bac	Reduces water intake and body weight by improving the morphology of intestinal mucosa	(Qi et al., 2019)
Colitis	Ginseng polysaccharide	228 mg/kg/day; 7 days	DSS-induced SD rats	Bif, Clo, Lep, Lac	Ent, Bac	Reduces anal prolapse, surrounding hyperemia by decreasing MPO activity, serum IL-1β, IL-6, TNF-α levels, and increasing IL-4, IL-10 levels.	(Shen et al., 2018)
Colitis	Ginseng polysaccharide	50-200 mg/kg	DSS-induced SD rats	Pro, All	Fir, Akk	Ameliorates intestinal mucosal tissue injury and intestinal inflammation by enhancing mTOR dependent autophagy, inhibiting NF-κB inflammatory pathway, and decreasing il-6, TNF-α and IL-8 inflammatory factors.	(Wang et al., 2021)
Colitis	Ginsenoside Rk3		C57BL/6JFandd mice	Bac, All, Bla	Fir	Repairs intestinal barrier dysfunction by increasing the expression of tight junction proteins (ZO-1, Occludin and Claudin-1), reducing colonic inflammatory cytokine levels, and suppressing TNF-α, IL-1β, and IL-6 overproduction.	(Bai et al., 2021)
Colitis	Ginsenoside Rk3	30 and 60 mg/kg/day; 56 days	HFD-C57BL/6 mice	Bac, Lac, Rum, Par, Mur, Pre, Ara, But	Fir, Pro, Hel, Clo, Akk, Ent, Ana, Ace	Inhibits the NF-κB pathway, the expression of McP-1, STAMP2, NADPH, TNF-α, TLR4 and increases the expression of ZO-1, Occludin and SCFAs to decrease the serum levels of TNF-α, IL-1β, IL-6, LPS.	(Chen et al., 2021)
Colitis	Red ginseng and coix seed	4 ml/kg; 14 days	TNBS-induced Wistar rats	Lac, Bif	Col	Improves bloody stools and body weight.by decreasing MCV and MCH and reducing DAI, MPO, MDA levels.	(Guo et al., 2015)

Mur, Muribaculaceae; Pre, Prevotellaceae; Ara, Arabidopsis; Allo, Alloprevotella; Prevo, Prevotae TNBS, trinitro-benzene-sulfonic acid.

### Colitis

Colitis is a chronic and persistent inflammatory environment leading to colon cancer, which is the third deadliest cancer of the world (Bocchetti et al., 2021). There are many factors leading to colitis, but the most fundamental is the gut microbiota. The changes in the composition and diversity of gut microbiota could lead to the damage of intestinal barrier and the immune function, and increase high levels of pro-inflammatory cytokines in the intestine and the incidence of erosion and ulcer (Hering et al., 2012). Studies have shown that probiotics supplementation can regulate bile acid metabolism and alleviate enteritis symptoms in mice (Liu and Wang, 2021). Probiotics can also restore unbalanced gut microbiota, enhance intestinal mucosal barrier function, improve intestinal immunity, and reduce gastrointestinal infection (Shen et al., 2018). Therefore, restoring the structure of gut microbiota is an important mechanism for the treatment of colitis. Ginseng has an anti-inflammatory and analgesic effects (Lee et al., 2019) and it could prevent the colitis and related cancer induced by dextran sodium sulfate (Shin et al., 2020). In the studies of dextran sodium sulfate (DSS)-induced SD rats, ginseng polysaccharides can significantly up-regulate the relative abundance of probiotics, reduce the relative abundance of pathogenic bacteria, and inhibit a variety of inflammatory signal pathways (Shen et al., 2018; Wang et al., 2021). Ginsenoside Rk3 can increase the abundance of probiotics such as *Bacteroides* and *Lactobacillaceae* and reduce the abundance of pathogenic bacteria that can promote the production of inflammatory factors such as *Proteobacteria*, *Helicobacteraceae*. At the same time, ginseng can decrease the ratio of *Firmicutes* and *Bacteroidetes*, increase the expressions of tight junction protein 1 (ZO-1) and occludin, improve the integrity of intestinal barrier and intestinal immune function through the inhibition of toll-like receptor 4 (TLR4)/NF-κB and other inflammatory pathways (Bai et al., 2021; Chen et al., 2021). The treatment of red ginseng and coix seed in the trinitrobenzene sulfonic acid-induced colitis rats is achieved by increasing the abundance of probiotics and reducing the abundance of pathogenic bacteria (Guo et al., 2015). In addition, ginsenoside Rg1 has been found could be transformed by gut microbiota in colitis model, this may the underling mechanism of anti-colitis effects of ginsenosides (Lee et al., 2015).Collectively, ginseng can inhibit the inflammation and improve colitis by reducing the relative abundance of pathogenic bacteria, such as *Helicobacter*, and increasing the relative abundance of *Lactobacillus* and *Bacteroides* probiotics (**Table 4**).

### Other Diseases

Importantly, many studies have proved that ginseng, ginsenosides, and ginseng polysaccharides play potential effects on other diseases. For male Wister rats with spleen deficiency syndrome, the combination of ginseng and wild jujube could up-regulate the relative abundance of *Firmicutes, Bacteroidetes, Lactobacillus*, and *Bifidobacterium*, reduce the relative abundances of *Actinobacteria*, *Proteobacteria*, *Streptococcus*, *Escherichia*, *Shigella*, *Veillonella*, and *Enterococcus*, reverse the pathological state of the gut microbiota imbalance of the spleen deficiency syndrome, and improve the spleen deficiency syndrome (Li et al., 2020). For the fatigue, two studies showed that the extracts of ginseng and fermented ginseng leaf can recover from exercise-induced fatigue by improving the structure of gut microbiota and reducing the level of inflammation (Zheng et al., 2021; Zhou et al., 2021). For neurological diseases, ginseng extract, ginsenoside or the formula containing ginseng can regulate gut microbiota to play neuroprotective effect and improve memory impairment. In the rat ischemia/reperfusion model, ginsenoside Rb1 can increase the relative abundance of *Lactobacillus* and gamma-aminobutyric acid receptors to reduce the proinflammatory cytokines for the improvement of neuroprotection (Chen et al., 2020). In the rats induced by D-galactose, ginseng decoction called Dushen Tang showed good curative ability for memory impairment by up-regulating *Bacteroidetes* and down-regulating *Lactobacillus* (Wang et al., 2021). In the mice with Parkinson’s disease, the treatment with the extract from KRG can improve neuronal function and alleviate Parkinson’s symptoms by up-regulating the relative abundance of *Eubacterium* and down-regulating the relative abundances of *Verrucomicrobia* and *Ruminococcus* (Jeon et al., 2020). For Alzheimer’s disease, a formula named Qisheng Wan containing ginseng and ginsenoside Rg1 can restore the structures of disordered gut microbiota and reduce the levels of inflammatory factors to reduce the symptoms of Alzheimer’s disease (Wang et al., 2020; Xiong et al., 2022). In the ApcMin/+ mice during the development of colorectal cancer, ginsenoside Rb3 and Rd can promote the growth of beneficial bacteria (*Bifidobacterium* spp., *Lactobacillus* spp., *Bacteroides acidifaciens*, and *Bacteroides xylanisolvens*) and lower the abundance of cancer cachexia-related bacteria (*Dysgonomonas* spp., *Helicobacter* spp.) to reinstate mucosal architecture and improve mucosal immunity (Huang et al., 2017). Moreover, ginsenosides can facilitate the therapeutic effect of cyclophosphamide in the mice with mammary carcinoma, which may be related with the increases of anti-tumor cytokines and the production of gut probiotics (*Akkermansia*, *Bifidobacterium*, and *Lactobacillus*) (Zhu et al., 2021). In addition, ginseng polysaccharides at 200 mg/kg can potentiate anti-tumor effect of anti-programmed cell death 1/programmed cell death ligand 1 immunotherapy in non-small lung cancer by regulating the relative abundance of bacteria such as *Escherichia*, *Rikenella, Parabacteroides distasonis*, and *Bacteroides vulgatus* (Huang et al., 2021).

The products of red ginseng, including its water extract, 50% ethanol extract and bifidobacterial-fermentation of ethanol extract were compared to explore their mechanisms against ovalbumin-induced allergic rhinitis. It found that bifidobacteria-fermented extract of red ginseng can reduce the levels of IL-4, IL-5, and IL-13 in the colon and restore the populations of gut microbiota (*Bacteroidetes*, *Actinobacteria*, and *Firmicutes*) (Kim et al., 2019). Furthermore, the same research group reported that bifidobacteria-fermented extract of red ginseng and its main constituent, ginsenoside Rd, protopanaxatriol can ameliorate gut dysbiosis (*Bacteroidetes* and *Proteobacteria* populations) to mitigate anxiety/depression (Han et al., 2020). Additionally, the intake of ginseng extract for 34 weeks can decrease the abundance of *Bifidobacteriaceae* and *Lactobacillus*, and increase the abundance of *Proteobacteria*, *Methylobacteriaceae*, and *Parasutterella, Sutterella*, which suggest that it regulate the host-gut metabolism in the normal rats (Sun et al., 2018). Collectively, ginseng can up-regulate the relative abundance of probiotics, reduce the relative abundance of pathogenic bacteria, or restore the structure of unbalanced gut microbiota to prevent and treat various diseases, including fatigue, neurological diseases, cancer, allergic rhinitis, and depression (**Table 5**).

**Table 5:** Summary of anti-other diseases activities of ginseng by regulating gut microbiota and related pathways.

Conditions	Compounds/Extracts	Dose/Period	Models	Gut microbiota		Mechanisms	Refs.
				Up	Down		
Spleen deficiency syndrome	Ginseng and Jujube seeds	0.4 mg/mL	Wistar rats	Fir, Bac, Lac, Bif	Act, Pro, Str, Esc, Shi, Ent, Vei	Reverse carbohydrate metabolism, signal transduction, and amino acid metabolism.	(Li et al., 2020)
Exercise-induced fatigue	Fermented ginseng leaf	50 mg/kg/d 28 days	SD rats	Bac, Ver, Def	Fir, Pro, Act,	Decreases hypoxanthine and isoprostane, increases protein expression of Pax7 and MyoD1, decreases gene expression of MyHC-I and MyHC-IIb, ameliorates the levels of acetic acid, propionic acid, total short chain fatty acids, TNF-α and IL-10.	(Zheng et al., 2021)
Sports fatigue	Ginseng extract	1.42 g/kg; 14 days	Exercise-induced fatigue SD rats	Bac, Lac, Bif, Str, Clo, Cop	Fir, Ana	Decreases the levels of IL-1β, LA, CP, LDH, BUN and GLU by inhibiting the expression levels of TUDCA, TCDCA, UDCA3, CDCA3 and TGR5, further regulating the metabolism of butyric acid and tryptophan.	(Zhou et al., 2021)
Neuroprotection	Ginsenoside Rb1	50 mg/kg; 13 days	PGF-SD rats	Lac		Increases the levels of A subunit and B subunit in GABA and IL-1β, IL-6, TNF-α to reduce neurological deficit and cerebral infarct area.	(Chen et al., 2020)
Memory impairment	Dushen Tang	0.3 g/kg/d; 49days	D-gal-induced SD rats	Bac	Lac	Inhibits nerve damage and exerts antiaging effects by activating the cAMP signaling pathway.	(Wang et al., 2021)
Parkinson’s disease	Korean red ginseng extract	100 mg.kg	MPTP-induced PD mice	Eub	Ver, Rum, Akk	Prevents MPTP-induced dopaminergic neuronal death, activation of microglia and astrocytes, and accumulation of α-synuclein in the SN, and the regulation of inflammation-related factors in the colon.	(Jeon et al., 2020)
Alzheimer’s disease	Qishen Wan formula (contain ginseng)	5.6-22.4 g/kg	SD rats	Akk, Lac, Bif	Ali, Lach	Reduces Aβ_1–42_ concentration and decreases the levels of NF-κB, TNF-α, and IL-6 to reduce pathological damage of hippocampus.	(Xiong et al., 2022)
Alzheimer’s disease	Ginsenoside Rg1	7.5-30 mg/kg; 56 days	Conventional tree shrews	Pro, Ent, Esc	Fir, Str, Lac, Pas	Inhibits the expression of Tau in hippocampus and cortex to improve learning and memory.	(Wang et al., 2020)
Colon cancer	Ginsenoside Rb3 and Rd	20 mg/kg; 56 days	Apc Min/+ mice	Lac, Bif, Rum, Bac, Des, Pre, Pse, Acid	Clo, Hel, But,Fae, Dys	Decreases the expression levels of iNOS, N-cadherin, FOXP3, CXCL10, and increases the expression levels of arginase-1 and TREm-2 to reduce serum IL-1β, IL-6, IL-12, IL-17 and IL-23 size and number.	(Huang et al., 2017)
Breast cancer	Ginsenoside and Cyclophosphamide	2,205 g ginsenosides extract/6000g ginseng crude drug, 1 h	ICR mice	Bif, Akk, Ver, Fir, Lac, Act, Ent	Bac, Lach, Odo, Pre	Inhibits the NF-κB pathway, promotes the expression of Caspase-3, Nrf2 and tight junction protein to reduce tumor volume and improve the pathological status of small intestine.	(Zhu et al., 2021)
Non-small cell lung cancers	Ginseng polysaccharides	200 mg/kg	PD-1 knock-in mice	Mur, Ery, Bur	Pre, Esc, Lac, Rik, Ach	Reduces tumor weight, increases the levels of IFN-γ, TNF-α, GZMB and the expression of CLCA3, TFF3, AGR2, Zg16, Pla2g10, Guca2a.	(Huang et al., 2021)
Allergic rhinitis	Fermented red ginseng and ginsenoside Rd	20 mg/kg; 30 days	BALB/c mice	Bac, Act	Fir	Promotes the expression of IgE and inhibits the myeloperoxidase activity to reduce eosinophils.	(Kim et al., 2019)
Anxiety and depression	Bifidobacterium fermented red ginseng and ginsenoside Rd	10-25 mg/kg/day; 5 days	2,4, 6-TNBS-induced C57BL/6 mice	Pro, Fir, Bac, Parap, Mur, Ali	Bac, Ent, Lach, Rum, Col	Inhibits the NF-κB pathway, the expression levels of BDNF and Myeloperoxidase activity to reduce IL-6, TNF-α and depressive behavior.	(Han et al., 2020)
Metabolism and gut microbial influence	Ginseng extract	100 mg/kg; 238 days	Wistar Rats	Bif, Lac, Meth, Para	TM7	Reduces the levels of IL-2, IL-6, IgG and IgM proinflammatory factors and increases the levels of IL-4, IL-10 and IgA anti-inflammatory factors by improving the immune system.	(Sun et al., 2018)

Vei, Veillonella; Meth, Methylobacteriaceae; Pas, Pasteurellaceae; Lep, leptum; Odo, Odoribacter Parap, Paraprevotella; Ali, Alistipes; Pse, Pseudomonas; Acid, Acidoides; But, Butyricimonas; Cam, Campylobacter; Dys, Dysgonomonas.

**Figure 1:**
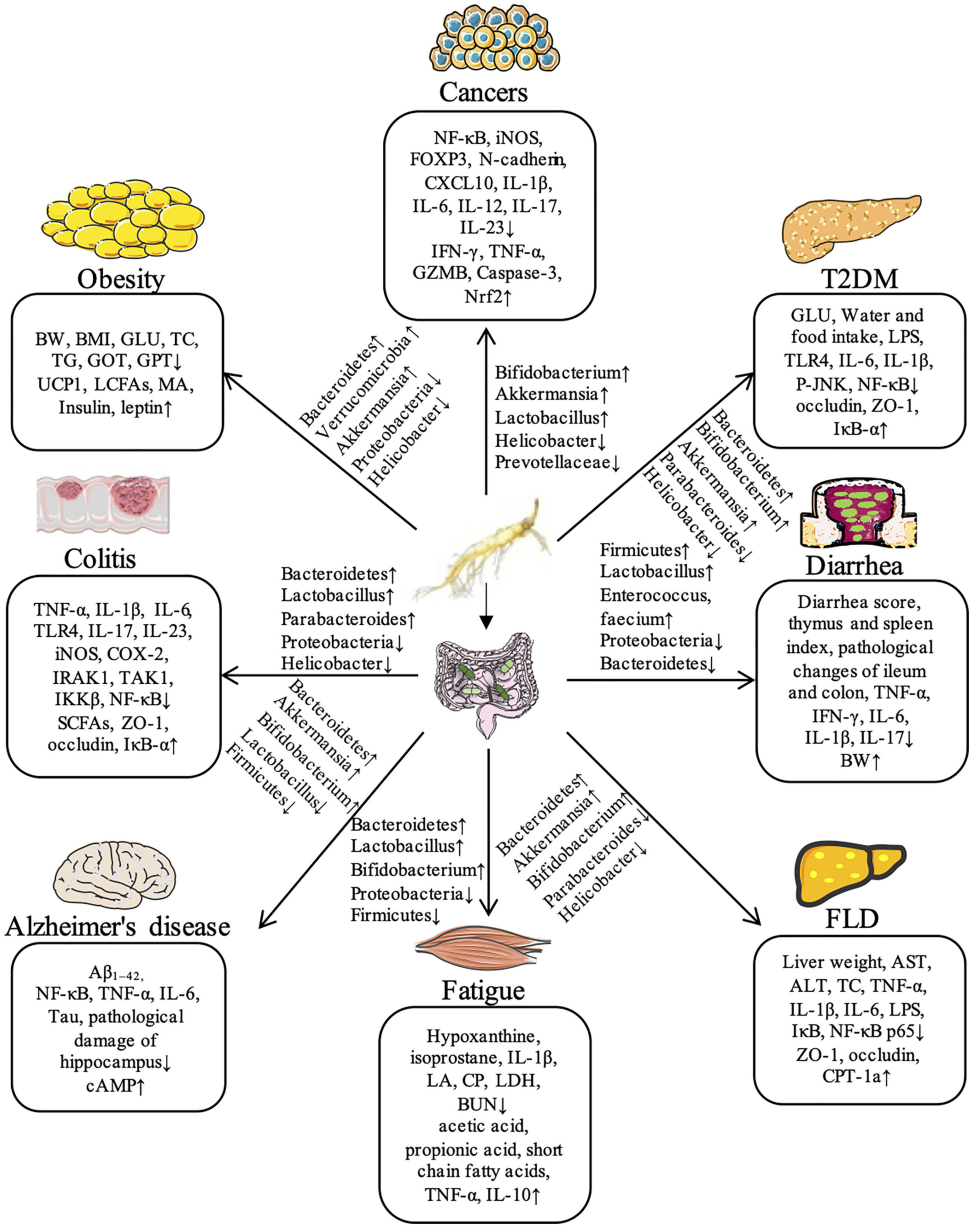
Ginseng shows its potential therapeutic effects on a variety of diseases through the regulation of gut microbiota in animal models.

In summary, ginseng has significant therapeutic effects on a series of diseases, including obesity, T2DM, liver diseases, colitis, diarrhea, exercise-induced fatigue, Alzheimer’s disease, and cancer by regulation gut microbiota. Recent studies demonstrate that ginseng can further inhibits NF-κB and other inflammatory pathways to reduce the levels of proinflammatory factors (TNF-α and IL-1β) and increases the expressions of key proteins such as TLR4, ZO-1, and Occludin by regulating the species diversity and relative abundance of intestinal flora, thus maintaining the intestinal mucosal barrier homeostasis. The flora regulated by ginseng mainly includes *Bacteroides*, *Bifidobacterium*, *Parabacteroides*, *Akkermansia*, *Helicobacter*, *Lactobacillus*, and *Proteobacteria* during the process of different diseases. The detailed information for the compositions of gut microbiota and regulated signaling pathways in the therapeutic effect of ginseng against various diseases has been shown in **Figure 1**.

## Transformation and Modification of Ginsenosides by Gut Microbiota

Microbiota is an important core to host metabolism, could regulate the expression and function of enzymes involved in drug metabolism (Xu et al., 2017), it can also participate in the regulation of ginsenoside transformation by producing nitrate reductase and glucuronidase to hydrolyze glycosidic bonds (Yang et al., 2020). Besides, many important components such as folate, indole, gamma-aminobutyric acid, and short chain fatty acids (SCFAs) are produced by the metabolism of ingested ingredients by microbiota. These metabolites also are the raw materials for the synthesis of above-mentioned molecules. Therefore, gut microbiota plays an vital role in the pharmacokinetics and pharmacodynamics of natural products (Pan et al., 2019).

**Figure 2:**
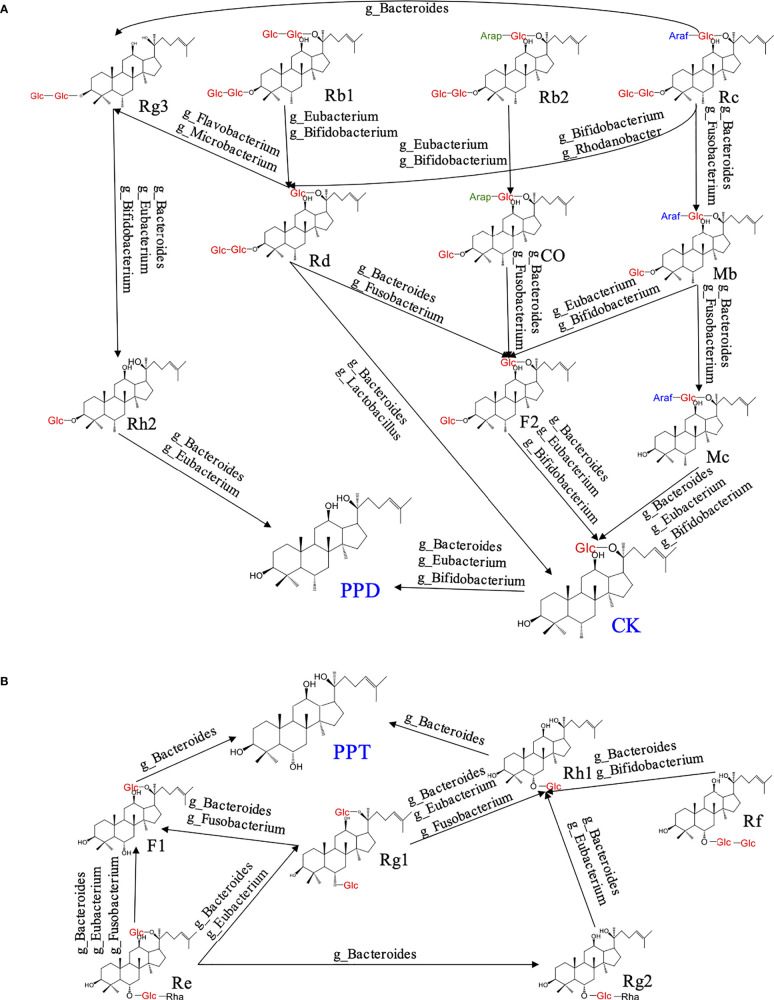
The metabolism of ginsenosides under the action of gut microbiota.

The oral utilization of ginsenosides is very low, and the activity of some ginsenosides is not good, hence they need to be transformed to improve their activity. Ginsenoside compound K (CK) is a kind of ginsenoside with good activity, which is transformed from a variety of ginsenosides through different processing. A large number of studies have found that ginsenoside CK has many pharmacological effects that it can induce apoptosis of colon cancer cells (Pak et al., 2020), treat lung cancer (Jin et al., 2018), and reduce the formation of atherosclerotic plaque (Zhou et al., 2016). Together, most studies have proved that CK has a significant therapeutic effect on inflammation and cancer. Structurally, CK has less Arabinofuranose (Araf), arabopyranose (Arap), and glucose (Glc) than the initial ginsenoside. Ginsenoside Rc can be transformed into Mb and Mc by removing Glc at C-3 under the action of *Bacteroides* and *Fusobacterium*, and then into CK by removing Araf at C-20 through *Bacteroides*, *Eubacterium* and *Bifidobacterium* (Choi et al., 2018). Ginsenoside Rb2 can first remove the Glc at C-3 through *Bifidobacterium* and *Eubacterium*, then remove the Araf at C-20 under the action of *Bacteroides* and *Fusobacterium* and convert it into F2, and finally transformed into CK under the action of *Bacteroides* and *Fusobacterium* (Kim et al., 2018). Ginsenoside Rb1 has only three more molecules of Glc than CK, which is transformed into CK under the action of *Eubacterium, Lactobacillus* and *Bacteroides* (Quan et al., 2013). In addition, Ginsenoside Rd and F2 are transferring states for CK transformation. Ginsenoside Rd is usually transformed from Rb1, Rb2 and Rc by removing redundant Araf and Glc under the action of *Eubacterium* and *Bifidobacterium*, while F2 is transformed from Mb, Co and Rd by removing Araf or Glc under the action of *Eubacterium*, *Fusobacterium* and *Bacteroides* (Zhang et al., 2021). Meanwhile, it found that ginsenoside which have Arap and Araf at C-20 preferentially removed Glc at C-3 during transformation. *Bacteroides* tends to preferentially remove the Glc at C-3. In addition, in the whole process of CK transformation, the enzymatic hydrolysis of Araf group mainly depends on *Bifidobacterium*, *Bacteroides* mainly guides the enzymatic hydrolysis of Glc, and the enzymatic hydrolysis of Arap is mainly mediated by *Fusobacterium* (**Figure 2A**).

Proto-ginsenoside of ginsenodiol type (PPD) has also been shown to have a significant inhibitory effect on breast cancer (Zhang et al., 2018), uterine cancer (Jo et al., 2021), prostate cancer (Cao et al., 2014), and metastasis of cancer cells (Lu et al., 2020). Although PPD has good pharmacological activity, it cannot be directly extracted from ginseng, it needs to be processed and transformed under the action of gut microbiota *in vivo*. Most of PPD is transformed from CK by removing the Glc at C-20 under the action of *Bacteroides*, *Eubacterium* and *Bifidobacterium*, and the other part is transformed from ginsenoside Rg3 by removing the two molecules of Glc at C-3 under the action of *Bacteroides*, *Eubacterium* and *Bifidobacterium* (**Figure 2A**). Proto-ginsenoside of ginsentriol type (PPT) is also a saponin with good activity, which has pharmacological effects such as reducing memory and cognitive impairment, improving Alzheimer’s disease (Lu et al., 2018), inhibiting septic shock and peritonitis (Jiang et al., 2020), improving cellular inflammation (Kim et al., 2015). PPT is not an initial saponin, which is transformed from a variety of ginsenoside under the action of gut microbiota in the intestine. In the whole transformation process of PPT, ginsenosides Re and Rf are the initial ginsenoside and ginsenoside Rh1 is the key secondary ginsenoside. During the transformation, ginsenoside Re can remove Glc or rhamnose (Rha) under the action of *Fusobacterium*, *Bacteroides* and *Eubacterium*, and then convert to F1, Rg1 and Rg2 (Bi et al., 2019). Ginsenoside Rh1, as a transferring style for PPT transformation, can be transformed from Rg1, Rg2, Rf by removing Glc, Rha and Glc under the action of *Bacteroides*, *Eubacterium* and *Bifidobacterium*, respectively. Under *Bacteroides*-mediated enzymatic hydrolysis, ginsenoside F1 and Rh1 can remove Glc at C-6 or C-20 to finally transformed into PPT (Chen et al., 2007). In addition, ginsenoside Rg1 can remove Glc, Rha at C-6 and then convert into F1 under the action of *Fusobacterium* and *Bacteroides* (Kim et al., 2011).

**Figure 3:**
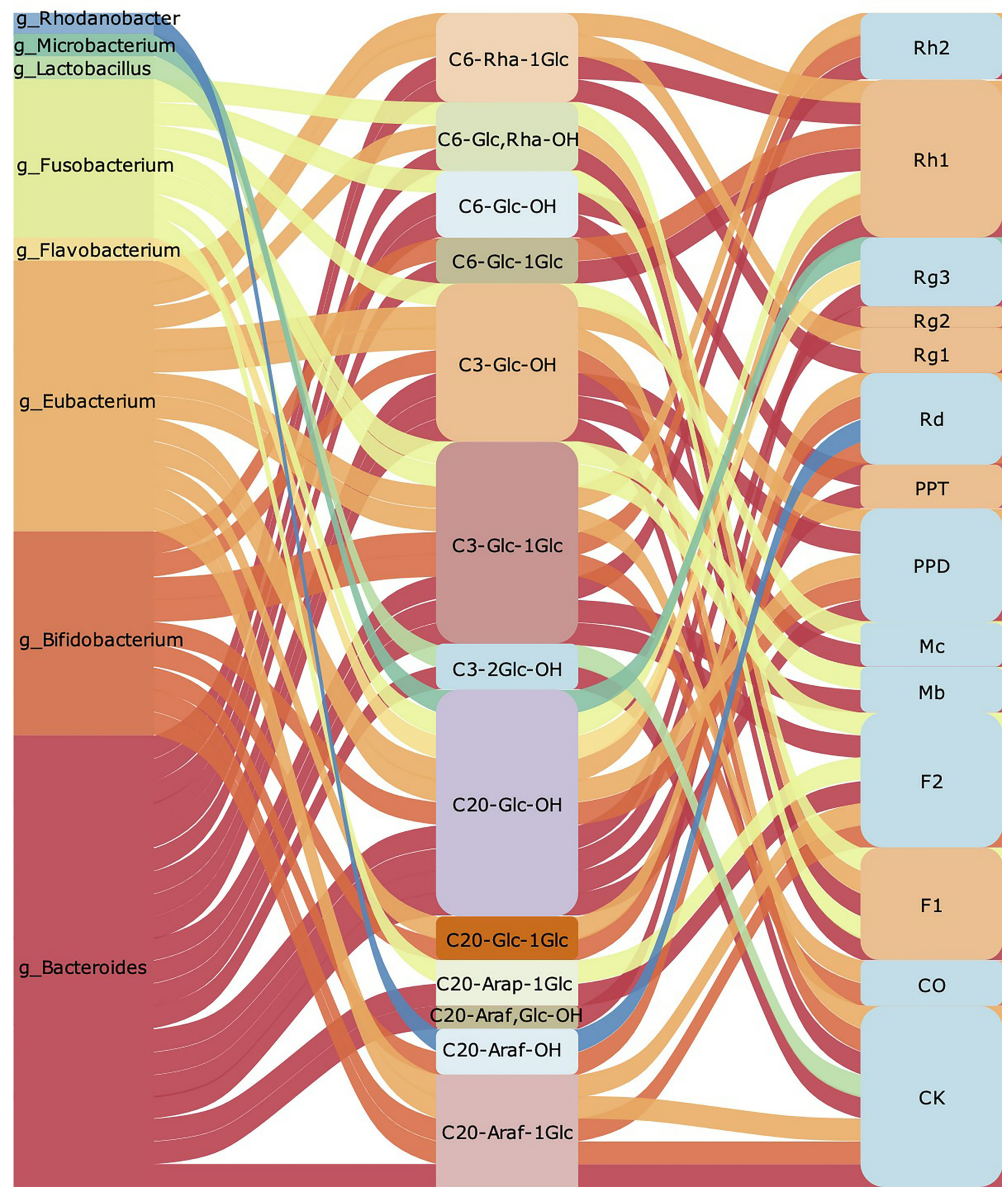
The summary of gut microbiota participates regulating ginsenoside transform. The left row is the gut microbiota involved in ginsenoside transformation. The middle part shows branched chain position of glycosidic bond hydrolysis, the removed sugar group, and the remaining functional groups respectively. The corresponding ginsenosides transformed are listed on the right row.

Summarily, we found that ginsenoside containing Rha such as Re, Rg2 could preferentially remove Rha when converted into PPT, and then hydrolyze other sugar groups. The hydrolysis of Rha for ginsenosides mainly depends on *Eubacterium* (**Figure 2B**). Besides, we concluded that *Bacteroides*, *Bifidobacterium* and *Eubacterium* are intestinal bacteria mainly involved in the transformation of ginsenoside. Among them, *Bacteroides* that degrade polysaccharides are most important bacteria. It has been found that *Bacteroidetes* could regulate thousands of enzymes, which can regulate specific carbohydrate degrading according to the structural information of the target glycan (Lapebie et al., 2019). As shown in **Figure 3**, we found that *Bacteroides* could induce the hydrolysis of glycosidic bonds at all possible positions. Besides, *Bifidobacterium* prefer to hydrolyze the glycosidic bond at position C-20, which mainly depends on its own glucosidase and glucanase, including β- glucan (Fernandez-Julia et al., 2021). Nowadays, it also has been approved that the hydrolysis by *Eubacterium is* closely related to β -glucosidase, but the further mechanism of ginsenoside transformation is unclear (Yang et al., 1995). According to the available evidence, *Lactobacillus* prefers to hydrolyze the glycosidic bond at position C-3, and the glycosidic bonds hydrolyzed by *Microbacterium* and *Rhodanobacterium* are more tended located at position C-20 (**Figure 3**). Collectively, *Bifidobacterium* and *Bacteroides* are the most beneficial bacteria and participate in the transformation process of ginsenosides *in vivo*. The therapeutic effect of ginseng depends on the further processing and modification of a variety of ginsenoside by the gut microbiota to enhance the absorption and the activity of ginsenoside in the body. At present, most researches of gut microbiota regulating ginsenoside transformation were still at an initial level, just showed the relationship between microbiota and transformation, but the internal mechanism and details need to be further clarified.

## Conclusion and Future Perspective

**Figure 4:**
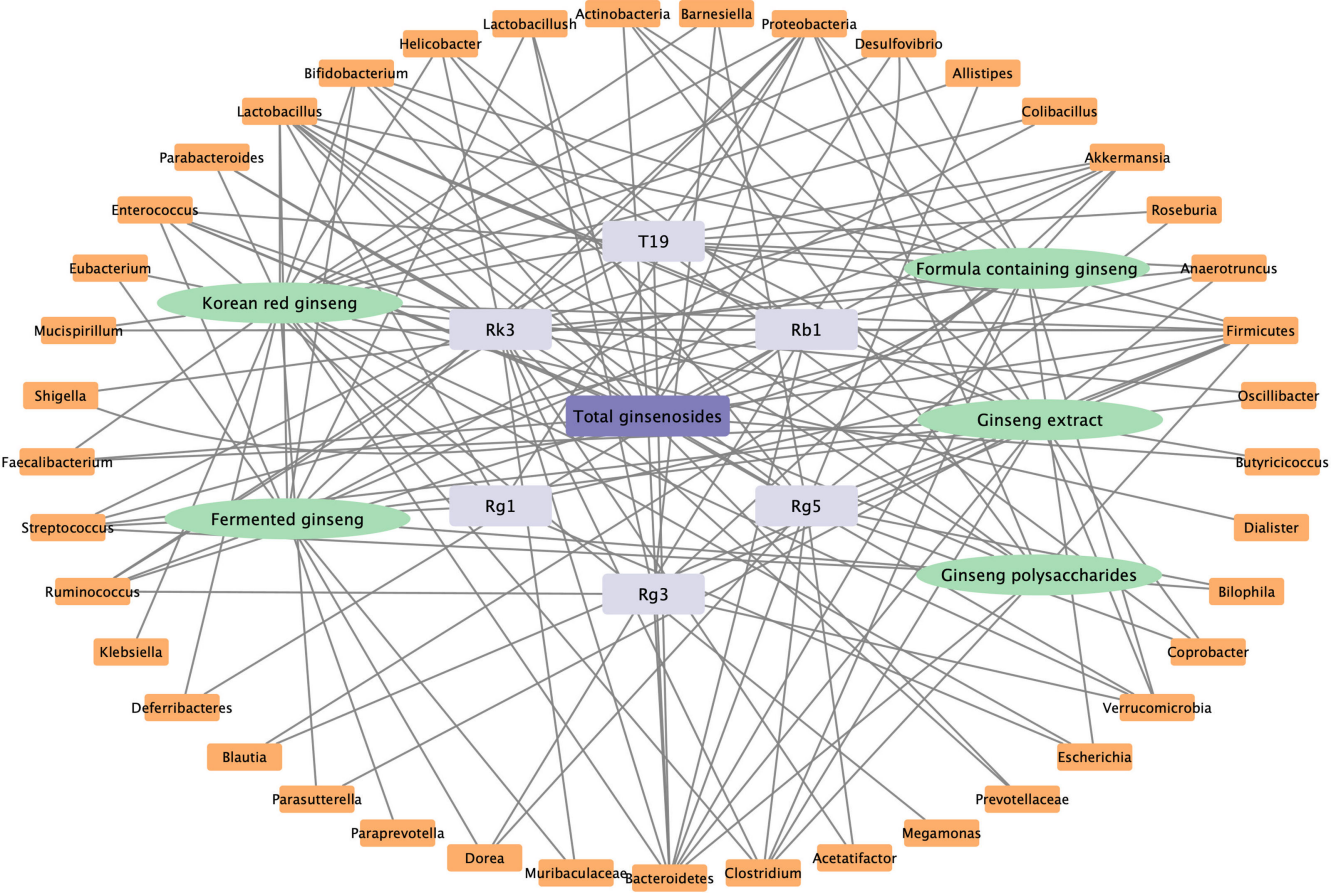
The correlation between ginseng and gut microbiota.

In our review, we summarized the findings of many studies, which showed that ginseng can play a role in the prevention and treatment of obesity, diabetes, fatty liver, colitis, diarrhea, cancer, and other diseases by regulating the host microbiota. At the same time, microbiota is also the key medium of ginsenoside transformation *in vivo*. The pharmacological functions of ginseng against various diseases might be related with the abundance and the structure of gut microbiota, including increase the relative abundance of probiotics (*Bacteroides*, *Lactobacillus*, *Bifidobacterium*, *Akkermansia*) and reduce the relative abundance of pathogenic bacteria (*Firmicutes*, *Helicobacter*, *Clostridium Proteobacteria*). Ginsenosides could be transformed into CK, PPD or PPT for better activities under the actions of probiotics, including *Bacteroides, Eubacterium, Bifidobacterium*, and *Fusobacterium*. Therefore, we can use probiotics combined with ginseng to improve the pharmacodynamic value of ginseng to fully play its pharmacological effect in future. Based on the current findings, the network relationship of ginseng extract, formula containing ginseng, ginsenosides, and polysaccharides with different microbiota have summarized and shown in **Figure 4**.

The following aspects and suggestions about ginseng and gut microbiota should be considered, according to the research status: (1) The number of human experiments is relatively small, and most experiments use animal models, very few the studies were performed in human. (2) The detection methods for gut microbiota are mostly limited to 16S rDNA sequencing, and the changes of microbiota are not revealed deeply and specifically enough. (3) Most of the current research results only find the changes of microbiota, but there are few reports on the regulation mechanism of microbiota. (4) The detection method is single, only one detection method is used, the method of multi-omics combination is not used. (5) The ginseng used in the current studies lacks standardized and unified standards. Researchers rarely report the quality control results of ginseng, which cannot ensure the repeatability and stability of the experiment. Therefore, we suggest that future researches should focus on the species, types, and strain of gut microbiota, their metabolites, and related signaling pathways regulated by ginseng. This review can provide better insight into the interaction of ginseng with gut microbiota in multiple disorders and ginsenoside transformation.

## Author Contributions

ZC and ZZ collected, analyzed, and reviewed the literature, wrote the main manuscript. JQL, HQ, QH, and JL assembled figures/tables. JQL, HQ, JL, JC, and QL added and checked references. ZZ, JM, and XL designed and supervised the manuscript. JM and XL revised the final version of the manuscript. All authors have read and agreed to the published version of the manuscript.

## Funding

This study was supported by the National Natural Science Foundation of China (U19A2013, 81804013), National Key Research and Development Program of China (2017YFC1702103), the Science and Technology Development Plan Project of Jilin Province (20200404057YY, 2020YFC0845000, 202002053JC, 2020122228JC).

## Conflict of Interest

The authors declare that the research was conducted in the absence of any commercial or financial relationships that could be construed as a potential conflict of interest.

## Publisher’s Note

All claims expressed in this article are solely those of the authors and do not necessarily represent those of their affiliated organizations, or those of the publisher, the editors and the reviewers. Any product that may be evaluated in this article, or claim that may be made by its manufacturer, is not guaranteed or endorsed by the publisher.

## Glossary

T2DM: type 2 diabetes mellitus
FLD: fatty liver disease
NF-κB: nuclear factor kappaB
NAFLD: Nonalcoholic fatty liver disease
TC: total cholesterol
TG: total triglyceride
SREBP-1c: sterol regulatory element binding protein-1c
FAS: fatty acid synthetase
ACC-1: acetyl-coenzyme A carboxylase 1
CPT-1a: carnitine palmityl transferase 1A
IκB: I-kappa-B
ZO-1: tight junction protein 1
TLR4: toll-like receptor 4
IL: interleukin
TNF-α: tumor necrosis factor-α
PPD: Proto-ginsenoside of ginsenodiol type
PPT: Proto-ginsenoside of ginsentriol type
Bif: Bifidobacterium
Fae: Faecalibacterium
Bla: Blautia
Efa: Efaecalis
Lac: Lactobacillus
Fir: Firmicutes
Bac: Bacteroidetes
Para: Parabacteroides
Ver: Verrucomicrobia
Akk: Akkermansia
Muc: Mucispirillum
Pro: Proteobacteria
Def: Deferribacteres
Hel: Helicobacter
Bar: Barnesiella
All: Allistipes
Osc: Oscillibacter
Cor: Coriobacteriaceae
Adl: Adlercreutzia
Dor: Dorea
Cop: Coprobacter
Par: Parasutterella
Clo: Clostridium
Fla: Flavonifractor
Pse: Pseudoflavonifractor
Ace: Acetatifactor
Bil: Bilophila
Ros: Roseburia
Sci: Scillibacter
Str: Streptococcus
Rum: Ruminococcus
Ana: Anaerotruncus
Ros: Roseburia
Aci: Acidobacteria
Des: Desulfovibrio
Ery: Erysipelothrix
Ver: Verrucomicrobia
Bla: Blautia
Wei: Weissella
Esc: Escherichia
Shi: Shigella
Kur: Kurthia
STZ: streptozocin
Eub: Eubacterium
Erys: Erysipelotrichaceae
Kle: Klebsiella
Meg: Megamonas
Dia: Dialister
Eps: Epsilonbacteraeota
Act: Actinobacteria
Deh: Dehalobacterium
Sut: Suterella
All: Allobaculum
Osci: Oscillospira
Pep: Peptostreptococcaceae
Col: Colibacillus
Ent: Enterococcus
Lach: Lachnospiraceae
Rik: Rikenella
Mur: Muribaculaceae
Pre: Prevotellaceae
Ara: Arabidopsis
But: Butyricicoccus
Allo: Alloprevotella
Prevo: Prevotae
TNBS: trinitro-benzene-sulfonic acid
Vei: Veillonella
Meth: Methylobacteriaceae
Pas: Pasteurellaceae
Lep: leptum
Odo: Odoribacter
Parap: Paraprevotella
Ali: Alistipes.
Pse: Pseudomonas
Acid: Acidoides
But: Butyricimonas
Cam: Campylobacter
Dys: Dysgonomonas
Bur: Burkholderiales
Ach: Acholeplasma
